# Genetic Predisposition for Dermal Problems in Hexavalent Chromium Exposed Population

**DOI:** 10.1155/2012/968641

**Published:** 2012-07-26

**Authors:** Priti Sharma, Vipin Bihari, Sudhir K. Agarwal, Sudhir K. Goel

**Affiliations:** ^1^Petroleum Toxicology Division, CSIR-Indian Institute of Toxicology Research (CSIR-IITR), Lucknow 226001, India; ^2^Epidemiology Division, CSIR-IITR, Lucknow 226001, India; ^3^Department of Biochemistry, Lucknow University, Lucknow 226007, India

## Abstract

We studied the effect of genetic susceptibility on hexavalent chromium induced dermal adversities. The health status of population was examined from the areas of Kanpur (India) having the elevated hexavalent chromium levels in groundwater. Blood samples were collected for DNA isolation to conduct polymorphic determination of genes, namely: *NQO1* (C609T), *hOGG1* (C1245G), *GSTT1,* and *GSTM1* (deletion). Symptomatic exposed subjects (*n* = 38) were compared with asymptomatic exposed subjects (*n* = 108) along with asymptomatic controls (*n* = 148) from a non contaminated reference community. Exposed symptomatic group consisted of 36.8% subjects who were *GSTM1* null genotyped as compared to asymptomatic where only 19.4% subjects were null. The exposed subjects with *GSTM1* null genotype were more susceptible to dermal adversities in comparison with wild genotyped subjects (OR = 2.42; 95% CI = 1.071–5.451). Age, smoking, gender or duration of residence were not found to have any confounding effect towards this association. Association with other genes was not statistically significant, nonetheless, possible contribution by these genes cannot be ruled out. In conclusion, variation in the polymorphic status of *GSTM1* gene may influence dermal outcomes among residents from Cr(VI) contaminated areas. Further studies are therefore, needed to examine these observations among different population groups.

## 1. Introduction

Chromium in trivalent form is a trace metal that humans require as an important bioelement for its exceptional role in metabolic processes [1]. On the other hand, the hexavalent form of Cr [Cr(VI)] is a toxic form and is reported to have deleterious health effects on the human beings. Cr(VI) has widespread applications in industries involved in leather tanning, manufacturing chrome sulfate, paints, dyes, and so forth. Occupational exposure to Cr(VI) is reported to cause contact dermatitis among workers [2]. Due to the improper ways of waste disposal from these industries, exposure to Cr(VI) is not limited at occupational environment but expands to areas in the vicinity of human residence [3–5].

Whereas there are extensive data available on the dermatological adversities due to occupational exposure of chromium compounds, only limited information is available concerning the risk and its mechanism involved following environmental exposure. Findings of the epidemiological studies on dermal outcomes among general populations have been equivocal. In a longitudinal health effects survey conducted by the Tokyo Metropolitan Government Bureau of Sanitation [6], an increase in incidence of contact dermatitis and eczema of the hands from residents in the contaminated areas compared to the controls was reported. In the contrary, self-reported-assessment-based study conducted among residents from a contaminated site at Glasglow found no evidence of harm to the health of residents [7]. The experimental studies also dictate remarkable variations in the behavior of chromium amongst individuals. Previous studies have reported large differences in the reduction of Cr(VI) to its lower oxidation states in human plasma and blood cells of different individuals [8–10] and chromium uptake in lymphoblastic cell lines derived from three different individuals [11]. In another study on chromium-sensitive subjects, dichromate evoked a positive patch test rate in only 8% of the subjects at 0.001% and 4% at 0.01% [12]. These observations point towards interindividual variability in response towards Cr(VI) exposure among the human beings.

Twin studies show that the genetic differences account for about a quarter of the variance in adult human lifespan. Genetic differences also contribute towards selection of genetically inherited tolerance among populations exposed to environmental toxicant [13]. With expanding involvement of genetic biodiversity in deciding biological response to various agents, it seems practical to consider the role of genetic factors towards health outcomes among general population exposed to Cr(VI). 

These genetic factors may correspond to those enzymes involved in the processes of Cr(VI) reduction inside the cell and the subsequent consequences [14]. So, by affecting the biological fate of Cr(VI) and its impact on various cell compartments, these genetic factors may influence the toxic impacts of Cr(VI). One out of these is NAD(P)H:quinone oxidoreductase (*NQO1*), also known as DT-diaphorase, reported to be involved in Cr(VI) reduction [15]. Existence of polymorphic forms of *NQO1* gene among human population is well documented. A transition of base C to T in the 609 codon of *NQO1* results in no detectable NQO1 activity [16] which may affect rate of Cr(VI) reduction. Following reduction of Cr(VI), the associated processes include generation of reactive oxygen species (ROS) leading to oxidative stress and DNA damage [17]. Prior studies have reported formation of 8-hydroxydeoxyguanosine (8-OHdG) base changes, an oxidation product due to occupational exposure of Cr(VI) [18, 19]. So, it is of interest to look for role of variation in 8-oxoguanine glycosylase gene (*hOGG1*) involved in the repair of 8-OHdG base changes [20]. A C to G nucleotide transversion at position 1,245 in exon 7 of the *hOGG1* gene is associated with the substitution of cysteine (Cys) for serine (Ser) at codon 326 which affects the biological activity of hOGG1 protein [21]. Further, glutathione S-transferases (GST), xenobiotic-metabolising enzymes are involved in the metabolic detoxification of various environmental carcinogens, oxidized lipid and DNA products generated by ROS-induced damage to intracellular molecules [22]. Two of the most relevant GST isoenzymes, *GSTM1 *(mu) and *GSTT1* (theta), are nonfunctional (due to deletion of a portion of gene) in appreciable percentage of human population. These deficiencies have been suggested to play an important role in cancer susceptibility [23–25]. Role of *GSTT1* and *GSTM1* polymorphism in relation to exposure towards other environmental toxicants has previously been demonstrated by our group [26, 27]. 

We understand that by affecting the individual's ability, genetic polymorphisms of *NQO1, hOGG1, GSTM1, *and* GSTT1* may influence occurrence of dermatological outcomes among general population exposed to Cr(VI). We conducted the present study which involved residents from Cr(VI) contaminated areas of Kanpur (Uttar Pradesh, India) [5]. An earlier health impact assessment-based study conducted by us revealed significantly higher prevalence of self-reports for dermal adversities among the residents from the contaminated areas as compared to residents with similar social and demographic features living in communities without elevated Cr(VI) levels. We hypothesize that the present study may explain the reason for differences in susceptibility towards Cr(VI) exposure among the residents.

## 2. Materials and Methods

### 2.1. Selection of Area

Kanpur, a city in Uttar Pradesh province of India, lies in Indo-Gangetic plain (26.4670° North and 80.3500° East). Reportedly, there are large numbers of leather tanneries and chrome sulphate manufacturing units located in and around Kanpur. Wastes from these industries are improperly disposed which has resulted in groundwater contamination at various areas of Kanpur [5]. Levels of Cr(VI) in groundwater have reached upto 124–258 times higher than the WHO permissible limit in some areas [5, 28, 29]. 

**Figure 1:**
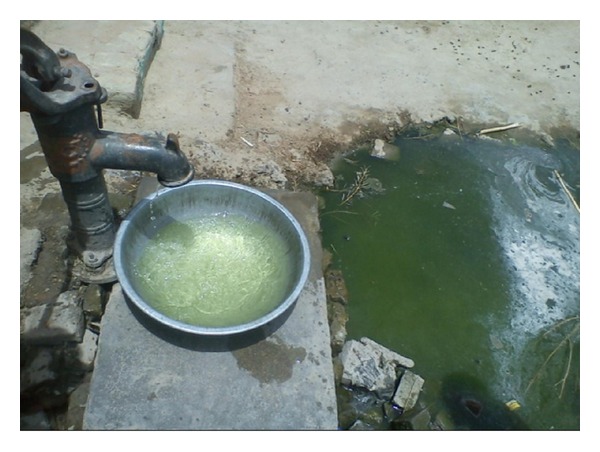
Photograph of yellow-colored contaminated water from a handpump.

We selected contaminated communities on the basis of previous reports by Central Pollution Control Board, UP. An area in the vicinity with no history of Cr(VI) contamination was also selected for a control population. To avoid any exposure misclassification, a standard diphenylcarbazide reagent method was used to estimate Cr(VI) in groundwater samples [30]. The estimated levels of Cr(VI) in groundwater from the contaminated area ranged from 8.0–38.4 ppm. The mean Cr(VI) concentration was 19.5 ± 9.4 ppm which was many folds above WHO permissible limit of 0.05 ppm.  Figure 1 shows photograph of contaminated yellow water from a handpump.

### 2.2. Selection of the Study Population

Subjects were recruited through the health camps. Ethical approval for the study was obtained from Institutional Human Ethics Committee of IITR. Before inclusion, written informed consent was obtained from all participants. Inclusion criteria of subjects was age equal to or more than 18 years, duration of residence not less than 5 years, no consumption of bottled water, and no present or past occupational exposure to Cr(VI). Occupational exposure to Cr(VI) includes various types of jobs which involve use or manufacture of Cr compounds for example, leather tanneries, cement, chrome sulphate manufacturing, paint and dye synthesis, chrome plating. Nonoccupational exposure to subjects is via environment for example, air, water, food chain. In our study, the exposure to subjects was predominantly through contaminated water. 

### 2.3. Health Examination and Record

A pretested questionnaire was used to gather demographic information. Heath information related with occurrence of dermatological symptoms, namely, itching or reddening of skin, flaky or scaly skin, and their specific histories were recorded in the questionnaire. A medical scientist also examined the subjects in accordance with recommendations outlined in the Declaration of Helsinki [31].

### 2.4. Blood Sample Collection

Blood samples (2-3 mL) were collected by venipuncture in EDTA-coated vacutainers (BD Biosciences), were stored at 4°C, and transported to the laboratory within 3-4 hours. 

### 2.5. Isolation of Genomic DNA

DNA was isolated from whole blood using commercial DNA isolation kit (Qiagen). Dissolved DNA was quantitated by optical density (OD) at 260 nm using Picodrop Spectrophotometer. DNA samples were stored at −80°C in small aliquots.

### 2.6. Genotyping for *GSTT1*, *GSTM1* Deletion Polymorphism

Analysis for *GSTT1* and *GSTM1* genetic polymorphism was done by multiplex PCR [32]. DNA (50 ng) was amplified in a 20 *μ*L reaction having 10 pmoles each of primers for *GSTT1*: 5′-TTCCTTACTGGTCCTCACATCTC-3′ and 5′-TCACGGGATCATGGCCAGCA-3′ and *GSTM1*: 5′-GAACTCCCTGAAAAGCTAAAGC-3′ and 5′-GTTGGGCTCAAATATACGGTGG-3′. As an internal control, exon 7 of the *CYPlAl* gene was coamplified using the primers: 5′-GAACTGCCACTTCAGCTGTCT-3′ and 5′-CAGCTGCATTTGGAAGTGCTC-3′. The PCR conditions consisted of an initial melting temperature of 94°C (5 min) followed by 35 cycles of melting (94°C, 2 min), annealing (59°C 1 min), and extension (72°C 1 min). A final extension step (72°C) of 10 min terminates the process. The PCR products from coamplification of *GSTT1*, *GSTM1, *and *CYPlA1* genes were then analyzed electrophoretically on 2% agarose gel. *GSTT1* and *GSTM1* wild genotypes yield band of 480 basepair (bp) and 215 bp, respectively, while no band is seen in case of deletion. *CYPlAl* gave band of size 315 bp in all the samples. 

### 2.7. Genotyping for *NQO1* (C609T) Polymorphism

Detection of C609T transition at gene *NQO1* was done using method reported by Harth et al. [33]. DNA (100 ng) was amplified using 10 pmoles of primers: 5′-GAGACGCTAGCTCTGAA CTGATT-3′ and 5′-AGCAAAATACAGATGGTGTCTCAT-3′. Thermal cycling conditions were firstly, 4 min at 94°C, 30 cycles of 1 min at 94°C, 1 min at 62°C, 1 min at 72°C and lastly 7 min at 72°C. PCR product (10 *μ*L) of 300 bp was digested with *HinfI *restriction enzyme (Fermentas) and run on 2.5% agarose gel. Wild genotype (C/C) showed one 280 bp band, heterozygous (C/T) showed 3 bands of 280 bp, 164 bp, and 116 bp and mutant genotype (T/T) showed 2 bands of 164 bp and 116 bp.

### 2.8. Genotyping for *hOGG1* (C1245G) Polymorphism

The C1245G transition at **h*OGG1* gene was detected using method reported by Wang et al. [34]. DNA was amplified using primers: 5′-AGGGGAAGGTGCTTGGGGAA-3′ and 5′-ACTGTCACTAGTCTCACCAG-3′. PCR consisted of an initial denaturation step for 5 min at 94°C, 35 cycles of denaturation for 20 s at 94°C, primer annealing for 20 s at 60°C, and primer extension for 40 s at 72°C, followed by a final extension step for 7 min at 72°C. PCR product (10 *μ*L) of size 200 bp was digested using *Fnu*4HI restriction enzyme (Fermentas) and run on 2.5% agarose gel. Heterozygous subjects (CG) exhibited two fragments (200 and 100 bp) while homozygous wild type (C/C) and mutant (GG) genotype exhibited single fragment of 200 bp and 100 bp, respectively.

### 2.9. Sequencing

The gene products for *GSTT1*, *GSTM1*, *NQO1* and *hOGG1* were sequenced. The amount of PCR product used in sequencing for *NQO1,* and *hOGG1* was 5 ng, while it was 7 ng for GSTM1 and 12 ng for GSTT1 [35].

### 2.10. Statistical Analysis

Descriptive statistics for exposed and control group were presented as mean and standard deviation. Frequencies and percentages were shown for categorical variables. Student's *t*-test and chi-square test were used to find out difference in distribution for socio-demographic variables among the two groups. Among exposed group, subjects afflicted with dermatological symptoms were regarded as “symptomatic” and the rest were designated as “asymptomatic”. Models were generated to analyze comparison among: (1) symptomatic exposed and asymptomatic control subjects; (2) symptomatic exposed and asymptomatic exposed subjects. Logistic regression procedures were used to estimate odds ratios (OR) with 95% confidence intervals (CI) to see influence of various genotypes on dermatological outcomes. Statistical significance was tested by chi-square test. Main predictor variables considered were genetic variants of the *GSTT1*, *GSTM1*, *NQO1,* and *hOGG1 *gene. Occurrence of the dermatological adversity (yes/no) was considered as outcome variable. The covariates analyzed were gender, age (≤35 and >35 years), smoking habit (never versus current or past), and duration of residence (≤20 and >20 years). Further, multivariate logistic regression analysis was also performed to adjust for confounding effect by the covariates. All the analyses were carried out using SPSS 13 (SPSS, Chicago, USA). A 0.05 cutoff point was set for the *P* value and applied in all the statistical analyses.

## 3. Results

### 3.1. Study Population and Its Characteristics

Study population comprised of a total of 146 exposed subjects (mean age ± SD: 36.29 ± 14.82 years) and 148 asymptomatic controls (mean age ± SD: 39.63 ± 14.39 years). Among exposed group, 26% (*n* = 38) subjects were having dermal problems, namely, itching, reddening, and crusting of skin. Duration of residence for the exposed subjects at the contaminated areas ranged from 2–79 years (mean ± SD: 24.17 ± 15.23 years). The sociodemographic characteristics of both the groups are shown in Table 1. Exposed population included 79 males and 67 females while control group consisted of 65 males and 83 females. Further, 8.8% subjects among control group and 19.2% among exposed group were smokers. There were differences in the number of smokers among two groups. So, we adjusted the risk estimates for any confounding effect by this variable.

### 3.2. Genotypic Distribution

**Figure 2:**
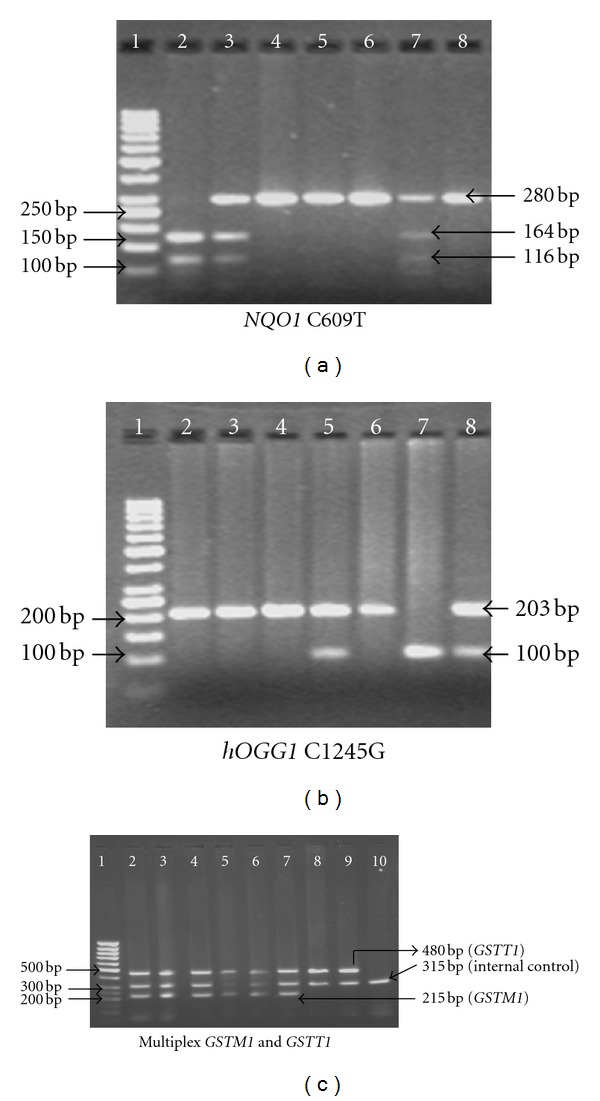
Representative gels of *NQO1* C609T, *hOGG1* C1245G and multiplex *GSTM1* and *GSTT1* genotyping.

Figure 2 shows the representative gel of *NQO1* C609T, *hOGG1* C1245G and multiplex *GSTM1* and *GSTT1* genotyping. Sequences of PCR products blasted with the reference gene from the database on National Centre for Biotechnology Information (NCBI) gave homology in the range of 96–99% (Table 2).

The genotypic distribution for *GSTT1* and *GSTM1, NQO1* and *hOGG1 *genesamong exposed and control group is shown in Table 3. Frequencies for *GSTT1* null and *GSTM1* null were 23.3%, and 24% among exposed population and 14.2%, and 37.8% among controls. Among exposed group, 38.3%, 51.1%, and 10.6% subjects were wild, heterozygous and mutant genotyped, respectively for *NQO1* C609T polymorphism and the respective frequencies among controls were 48.6%, 37.2%, and 14.2%. For *hOGG1* gene, respective frequencies for wild, heterozygous and mutant genotype were 47.3%, 41.1%, and 11.6% among exposed subjects and 36.5%, 53.4%, and 10.1% among controls. Hardy Weinberg equilibrium test showed accordance for *NQO1* and *hOGG1* gene in both population groups. The Hardy Weinberg equilibrium could not be tested for *GSTT1* and *GSTM1* because of the inability of the present PCR protocol to separate heterozygous carriers of the deletion polymorphisms.

### 3.3. Association between Genetic Polymorphism and Occurrence of Dermal Problems

We compared the distribution for various covariates and genetic polymorphic status of *GSTT1* and *GSTM1, NQO1* and *hOGG1 *genes among symptomatic exposed (*n* = 38) and asymptomatic control group (*n* = 148). It showed no significant difference in distribution among males versus females and subjects with age ≤35 versus age >35 years (Table 4). However, smoking was found to act as a confounding factor. Exposed group consisted of greater number of smokers (21%) as compared to controls (8.8%) and exposed smokers had higher prevalence of dermal problems as compared to control nonsmokers (OR = 2.77; 95% CI = 1.055–7.272). The difference in the distribution of genetic polymorphic status for *GSTT1* gene was found significant with respect to occurrence of dermal complaints. As compared to asymptomatic control group where 14.2% subjects were null genotyped, symptomatic exposed group was having 29% subjects having *GSTT1* null genotype. Odds ratio showing prevalence of dermal problems among *GSTT1* null genotyped as compared to wild genotyped subjects was 2.46 (95% CI = 1.064–5.705). However, after adjustment for smoking, influence of *GSTT1* polymorphism did not remain significant, although the odds ratio (OR = 2.53) did not change much. Further, there were no significant differences in distribution for *NQO1* (OR = 1.18), *hOGG1* (OR = 0.905) and *GSTM1* (OR = 0.96) gene among these groups.

Table 5 depicts distribution of covariates and genetic polymorphic status among symptomatic exposed (*n* = 38) and asymptomatic exposed group (*n* = 108). It was observed that the occurrence of dermal problems among exposed group was independent of gender, agecategory (≤35 versus >35 years), smoking status (never versus current or past), and duration of the residence (≤20 years versus >20 years). Analysis with genetic polymorphic status showed significant difference in distribution for *GSTM1* genotypes. Exposed symptomatic group consisted of 36.8% subjects who were *GSTM1* null genotyped as compared to asymptomatic where only 19.4% subjects were null. Odds ratio showed higher prevalence of dermal problems in null genotyped subjects compared to wild genotyped subjects (OR = 2.42; 95% CI = 1.071–5.451). The association with other genes, although, did not reach statistically significant level, however, higher odds ratios (OR > 20) were found in case of both *hOGG1* mutant (OR = 2.07; 95% CI = 0.96–4.469) and *GSTT1* null genotype (OR = 1.51; 95% CI = 0.651–3.484) compared to their respective wild genotypes. On the contrary, *NQO1* genetic polymorphism showed reverse association and subjects with null genotype had lower prevalence for dermal problems as compared to those having wild genotype (OR = 0.71; 95% CI = 0.329–1.529).

## 4. Discussion

In our previous studies on the population residing in Cr(VI) contaminated areas, higher prevalence of self-reports for dermal complaints, namely, itching, reddening, and scaling of skin was observed with higher mean Cr levels (approximately 6 folds) in hair of these residents compared to controls having similar sociodemographic status (unpublished observations). However, we noticed that not all residents were at risk to the dermal outcomes. Therefore, an attempt has been made to find out genetic linkages, if any, in causing wide variability in the outcomes among Cr(VI) exposed individuals. The general population living in hexavalent chromium-contaminated areas of Kanpur provided us with the opportunity to explore the role of genetic variants in causing variable response towards dermal problems among exposed individuals and also in comparison with unexposed individuals. 

In the present study on genetic variability, we found significant modification in the risk by *GSTM1* genetic polymorphism. It was observed that absence of *GSTM1* gene activity among residents from Cr(VI) contaminated areas was acting as a predisposing factor towards occurrence of dermal problems. This is suggestive of the role of *GSTM1* gene in the pathogenesis of dermal problems among subjects exposed to Cr(VI). 

Literature reports that a number of skin diseases are associated with oxidative stress [36] with a very specific role of *GSTM1* in protection against oxidative stress [37]. *GSTM1* catalyzes the conjugation of DNA hydroperoxides, namely, 4-hydroxynonenal, linoleic acid hydroperoxide which are mutagenic and cytotoxic product of lipid peroxidation [37]. Further, 5-hydroxymethyluracil, a mutagenic compound that is formed by either oxidative attack on the methyl group of the thymine base of DNA or from deamination of products formed by oxidation of 5-methylcytosine was also taken care by *GSTM1* [38]. Skin allergies are more common among individuals with genetic absence of *GSTM1* [39]. Arsenic-induced skin lesions [40] and non-melanoma carcinoma risk [41] are also reported in association with *GSTM1* null genotype. Cr(VI) induces carcinogenesis via oxidative stress pathway [42]. The role of glutathione (GSH) in Cr(VI) reduction inside the cell has also been highlighted [43]. Increased frequency of sister chromatid exchange (SCE) among Cr workers with *GSTM1* null genotype as opposed to nonnull genotype individuals is observed [44]. 

There are different distribution patterns of *GSTM1* null genotype among different ethnic groups which ranges from 23% to 48% in African populations, 33% to 63% in Asian populations, 39% to 62% in European populations, and 23% to 62% in U.S. populations [45]. Further, interindividual as well as interethnic variations alongwith toxicants exposure to the population across the world might be showing interindividual variations in the toxicant effect relationship. 

The genetic associations with other genes involved in Cr metabolism and disposition were not significant; however, this indicates possible influence of such genetic polymorphisms on the exposed population. Formation of 8-hydroxy deoxyguanosine has been demonstrated on occupationally exposed workers [18, 19]. In another study involving school children from communities near thermal power plant, greater urinary 8-OHdG concentration was found among children having elevated urinary chromium levels than those with lower urinary chromium [46]. Association of *GSTT1* genetic polymorphism with dermal manifestations [47] and its influence in causing variability among chrome plating workers is also described [48]. The role of *NQO1 *in cellular mechanism of Cr(VI) reduction has also been introduced [15]. Advocating the role of *NQO1* in Cr(VI) reduction raises possibility of increased production of lower oxidation state of chromium which is more toxic and concomitant production of ROS [14]. So, the compromised activity of *NQO1* due to genetic polymorphism may give protection from damage caused by Cr(III) production inside the cell. This might be the cause behind higher prevalence of dermal outcomes among *NQO1* wild genotyped subjects. 

Further, elicitation of health outcomes among human population depends not only on exposure conditions on which humans have no control, but also, on factors such as smoking for which a personal choice exists. Smoking is strongly associated with numerous dermatologic conditions including squamous cell carcinoma and psoriasis, although, the evidence linking smoking and melanoma, eczema, and acne is inconclusive [49]. Synergistic effect between arsenic exposure and tobacco smoking on risk of skin lesionswas reported by Chen et al. [50]. We also observed significant influence of smoking on the dermal outcomes, in association with environmental exposure to Cr(VI). Thus, we understand that smoking status of subject should be taken into consideration while determining health risk due to a toxic environmental agent. 

We accept that genetic research is not applicable for direct public health purposes, as it currently stands. Moreover, the ethical issues on revealing personal genetic information are also considerable. However, such studies can be worthwhile for the investigation of disease mechanisms, to give insights on potential therapies or to discover unidentified etiological agents involved in the case of diseases whose etiology is still unknown. These genetic studies are of high relevance for populations being chronically exposed to a toxic agent under low concentrations, a usually common scenario within residential settings. 

## 5. Conclusion

The present study reports that *GSTM1* genetic polymorphism may cause individuals bearing high-risk genotype more susceptible towards Cr(VI) exposure associated dermal outcomes. This could well explain why environmental exposures have aggravated effects, if they occur in a population of vulnerable subjects. However, more studies on role of genetic polymorphisms in association with Cr(VI) exposure among different populations are needed. With the increasing Cr(VI) toxic burden in vicinity of the human habitat, unraveling the role of such factors involved in modulation of toxic response is highly needed. Knowledge gained, thus, may not only help in screening high-risk groups but, in the long run, may also pave the path for personalized therapeutics measures. 

## Figures and Tables

**Table 1:** Demographic characteristics of the study participants.

Characteristics	Category	Exposed group	Control group	*P* value
		(*N* = 146)	(*N* = 148)	
Mean age	Mean ± SD	36.29 ± 14.82	39.63 ± 14.39	0.086^a^
Number of subjects	Males	79 (54)	65 (44)	0.0805^b^
	Females	67 (46)	83 (56)	
Smoking status	Smokers	28 (19.2)	13 (8.8)	0.0101^b^
Symptom category	Symptomatic	38 (26)	—	

SD: standard deviation.

Number in parenthesis denotes percentage.

^a^ *P* value for continuous variables calculated using Student's *t*-test.

^b^ *P* value for categorical variables calculated using Chi-square test.

**Table 2:** Summary of accession number of individual genes and their products sequenced after purification with percentage homology.

Gene	Accession number	Product	Product RFLP	Homology (%)
*GSTT1*	NM_000853	480 bp	—	96.6
*GSTM1*	NM_000561	215 bp	—	97.3
*NQO1* C609T	NM_000903	300	280, 164, 116	99
*hOGG1* C1245G	NM_016819.3	200	100 bp	99

**Table 3:** Genotypic distribution for *GSTT1*, *GSTM1*,* NQO1* C609T, and *hOGG1* C1245G genes among exposed and control group.

Characters	Category	Exposed group	Control group
		(*N* = 146) *n* (%)	(*N* = 148) *n* (%)
*GSTT1*	Wild	112 (76.7)	127 (85.8)
	Null	34 (23.3)	21 (14.2)

*GSTM1*	Wild	111 (76)	92 (62.2)
	Null	35 (24)	56 (37.8)

*NQO1*	Wild (C/C)	54 (38.3)	72 (48.6)
	Heterozygous (C/T)	72 (51.1)	55 (37.2)
	Mutant (T/T)	15 (10.6)	21 (14.2)

*hOGG1*	Wild (C/C)	69 (47.3)	54 (36.5)
	Heterozygous (C/G)	60 (41.1)	79 (53.4)
	Mutant (G/G)	17 (11.6)	15 (10.1)

Data for some samples are missing due to limited sample volume.

**Table 4:** Influence of various covariates and genotypes on dermatological adversities among exposed group compared with asymptomatic control group.

Characters	Category	Exposed SYMP	Control ASYMP	OR (95% CI)
		(*N* = 38) *n* (%)	(*N* = 148) *n* (%)	
Sex	Males	20 (52.6)	65 (43.9)	1
	Females	18 (47.4)	83 (56.1)	0.71 (0.345–1.441)

Age (years)	≤35	20 (52.6)	69 (46.6)	1
	>35	18 (47.4)	79 (53.4)	0.67 (0.408–1.109)

Smoking	Never	30 (79)	135 (91.2)	1
	Current/past	8 (21)	13 (8.8)	2.77 (1.055–7.272)^∗^

*GSTT1*	Wild	27 (71)	127 (85.8)	1
	Null	11 (29)	21 (14.2)	2.46 (1.064–5.705)^∗^
				2.53 (0.946–6.77)^#^

*GSTM1*	Wild	24 (63.2)	92 (62.2)	1
	Null	14 (36.8)	56 (37.8)	0.96 (0.458–2.005)

*NQO1*	Wild (C/C)	16 (44.4)	72 (48.65)	1
	Mutant (C/T + T/T)	20 (55.6)	76 (51.35)	1.18 (0.569–2.463)

*hOGG1*	Wild (C/C)	13 (34.2)	54 (36.5)	1
	Mutant (C/G + G/G)	25 (65.8)	94 (63.5)	0.905 (0.428–1.915)

SYMP: symptomatic, ASYMP: asymptomatic, OR: odds ratio, CI: confidence interval; ^#^odds ratio adjusted for smoking; ^∗^ *P* < 0.05.

**Table 5:** Influence of various covariates and genotypes on dermatological adversities among symptomatic exposed compared with asymptomatic exposed group.

Characters	Category	Exposed SYMP	Exposed ASYMP	OR (95% CI)
		(*N* = 38) *n* (%)	(*N* = 108) *n* (%)	
Sex	Males	20 (52.6)	59 (54.6)	1
	Females	18 (47.4)	49 (45.4)	1.08 (0.517–2.273)

Age (years)	≤35	20 (52.6)	61 (56.5)	1
	>35	18 (47.4)	47 (43.5)	1.17 (0.556–2.453

Smoking	Never	30 (79)	88 (81.5)	1
	Current/past	8 (21)	20 (18.5)	1.17 (0.468–2.94)

Duration of	≤20	19 (50)	62 (57.4)	1
residence (years)	>20	19 (50)	46 (42.6)	1.35 (0.642–2.83)

*GSTT1*	Wild	27 (71)	85 (78.7)	1
	Null	11 (29)	23 (21.3)	1.51 (0.651–3.484)

*GSTM1*	Wild	24 (63.2)	87 (80.6)	1
	Null	14 (36.8)	21 (19.4)	2.42 (1.071–5.451)^∗^

*NQO1*	Wild (C/C)	16 (44.4)	38 (36.2)	1
	Mutant (C/T + T/T)	20 (55.6)	67 (63.8)	0.71 (0.329–1.529)

*hOGG1*	Wild (C/C)	13 (34.2)	56 (51.8)	1
	Mutant (C/G + G/G)	25 (65.8)	52 (48.2)	2.07 (0.96–4.469)

SYMP: symptomatic, ASYMP: asymptomatic, OR: odds ratio, CI: confidence interval; ^∗^ *P* < 0.05.
